# Application of AI in Sepsis: Citation Network Analysis and Evidence Synthesis

**DOI:** 10.2196/54490

**Published:** 2024-04-15

**Authors:** MeiJung Wu, Md Mohaimenul Islam, Tahmina Nasrin Poly, Ming-Chin Lin

**Affiliations:** 1 Graduate Institute of Biomedical Informatics College of Medical Science and Technology Taipei Medical University Taipei Taiwan; 2 Department of Nursing Wan Fang Hospital Taipei Medical University Taipei Taiwan; 3 Department of Outcomes and Translational Sciences College of Pharmacy The Ohio State University Columbus, OH United States; 4 Taipei Neuroscience Institute Taipei Medical University Taipei Taiwan; 5 Department of Neurosurgery, Shuang Ho Hospital Taipei Medical University Taipei Taiwan

**Keywords:** AI, artificial intelligence, bibliometric analysis, bibliometric, citation, deep learning, machine learning, network analysis, publication, sepsis, trend, visualization, VOSviewer, Web of Science, WoS

## Abstract

**Background:**

Artificial intelligence (AI) has garnered considerable attention in the context of sepsis research, particularly in personalized diagnosis and treatment. Conducting a bibliometric analysis of existing publications can offer a broad overview of the field and identify current research trends and future research directions.

**Objective:**

The objective of this study is to leverage bibliometric data to provide a comprehensive overview of the application of AI in sepsis.

**Methods:**

We conducted a search in the Web of Science Core Collection database to identify relevant articles published in English until August 31, 2023. A predefined search strategy was used, evaluating titles, abstracts, and full texts as needed. We used the Bibliometrix and VOSviewer tools to visualize networks showcasing the co-occurrence of authors, research institutions, countries, citations, and keywords.

**Results:**

A total of 259 relevant articles published between 2014 and 2023 (until August) were identified. Over the past decade, the annual publication count has consistently risen. Leading journals in this domain include *Critical Care Medicine* (17/259, 6.6%), *Frontiers in Medicine* (17/259, 6.6%), and *Scientific Reports* (11/259, 4.2%). The United States (103/259, 39.8%), China (83/259, 32%), United Kingdom (14/259, 5.4%), and Taiwan (12/259, 4.6%) emerged as the most prolific countries in terms of publications. Notable institutions in this field include the University of California System, Emory University, and Harvard University. The key researchers working in this area include Ritankar Das, Chris Barton, and Rishikesan Kamaleswaran. Although the initial period witnessed a relatively low number of articles focused on AI applications for sepsis, there has been a significant surge in research within this area in recent years (2014-2023).

**Conclusions:**

This comprehensive analysis provides valuable insights into AI-related research conducted in the field of sepsis, aiding health care policy makers and researchers in understanding the potential of AI and formulating effective research plans. Such analysis serves as a valuable resource for determining the advantages, sustainability, scope, and potential impact of AI models in sepsis.

## Introduction

Sepsis is a life-threatening medical emergency [1] affecting approximately 48.9 million individuals globally each year and potentially contributing to over 11 million deaths [2]. Previous studies indicated that sepsis-related hospitalization can result in fatal outcomes in 30%-50% of cases [3,4]. However, prompt stratification and the timely administration of specific treatments have the potential to lower sepsis-related mortality. Identifying sepsis at an early stage can be challenging due to the complex pattern of the disease [5,6] and the diversity of the septic population [7].

Artificial intelligence (AI) has piqued interest in its excellent potential to stratify patients with a high risk of sepsis [8]. In recent times, AI models have seen widespread application in the prediction of sepsis and have shown superior performance compared with conventional statistical methods [9,10]. Yet, no study has shed light on the variety of AI applications and their potential and limitations in sepsis through a scientific consolidation of knowledge. Bibliometric analysis aids researchers in comprehending specific research fields, a crucial aspect for guiding both future research endeavors (eg, what else should we know) and practical implementation (eg, what should we do) [11]. This research aims to address the following questions, with the intent of advancing the previous research on the application of AI in sepsis: (1) What countries, institutions, sources, and documents have demonstrated the highest productivity within the realm of AI applied to sepsis? (2) What are the hot research topics and themes of research in the application of AI in sepsis? (3) What methods are mainly applied in the existing body of literature? (4) What types of limitations appeared in the existing literature regarding the application of AI in sepsis? and (5) What are the literature gaps and future research agendas?

In this study, we could systematically investigate shifts in publication growth, offering more valuable insights to fellow researchers and policy makers engaged in priority setting and assessment.

## Methods

### Data Source

We leveraged extracted data from the Web of Science Core Collection as of August 31, 2023. We used Web of Science for its comprehensive coverage across multiple databases, comprising a wide range of bibliometric indicators and literature from various disciplines. Using a predefined search strategy, we intended to include all relevant literature for bibliometric analysis. We used the following key words: *artificial intelligence* OR *computational intelligence* OR *deep learning* OR *computer aided* OR *machine learning* OR *support vector machine* OR *data learning* OR *artificial neural network* OR *digital image* OR *convolutional neural network* OR *evolutionary algorithms* OR *feature learning* OR *reinforcement learning* OR *big data* OR *image segmentation* OR *hybrid intelligent system* OR *hybrid intelligent system* OR *recurrent neural network* OR *natural language processing* OR *Bayesian network* OR *Bayesian learning* OR *random forest* OR *evolutionary algorithms* OR *multiagent system* AND *sepsis.* The collected records contained essential attributes, including publication date, authorship, institutional affiliation, geographic origin, and cited references. This data set served as the foundation for our subsequent analytical investigations.

### Inclusion and Exclusion Criteria

The titles and abstracts underwent initial screening by 2 independent authors (MW and TNP). If there was uncertainty from one reviewer regarding whether the article met the inclusion criteria, it was included for a thorough full-text review. Following this, both authors independently assessed the full text, and any differences in opinion were resolved through consensus with the research team. We considered studies for inclusion if they met the following criteria: (1) they were written in English, and (2) they applied AI models in the context of sepsis. In this study’s screening process, we included research or review articles published in peer-reviewed journals, conference proceedings, reviews, and early access articles. We excluded studies if they were published as letters, editorials, book chapters, or books.

### Data Collection and Preprocessing

To ensure compatibility with Bibliometrix and VOSviewer [12], we saved the data in the “**.txt” format, a format recognized by both tools for conducting analyses. Our data set encompasses a comprehensive range of information, including titles, list of authors, name of countries, list of institutions, abstracts, keywords, name of journals, and publication dates.

### Statistical Analysis

Bibliometrix and VOSviewer tools were used to uncover the knowledge structure, most influential countries, research hot spots, and productive authors, along with various bibliometric insights. The processed data were uploaded into these bibliometric tools, and analysis was conducted based on the information included within the data documents [13]. Afterward, we generated network maps among journals, authors, countries, and institutions, where individual points symbolized authors, countries, or institutions. Moreover, connected lines in the network maps depicted the relationships between these entities. Larger points and more robust lines indicated a higher number of articles and more substantial collaborative relationships, respectively [14,15].

We computed the annual growth rate of publications. The annual publication count, annual growth, and average growth rate of publications were determined through the following methods:



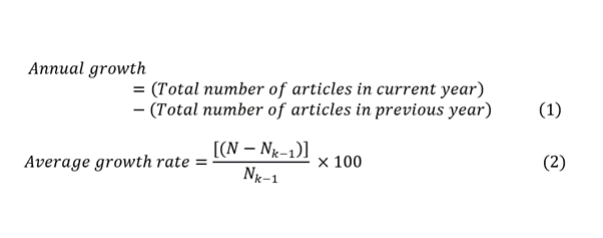



Where *N* is the total number of articles in the current year, and *N*_k–1_ is the total number of articles in the previous year.

Furthermore, we conducted an analysis of publication trends based on the following criteria: the top 10 most prolific countries, institutions, journals, authors, and studies in this area. The rankings of countries, institutions, journals, and authors were determined based on the number of articles.

## Results

### Distribution of Articles by Publication Year

**Figure 1:**
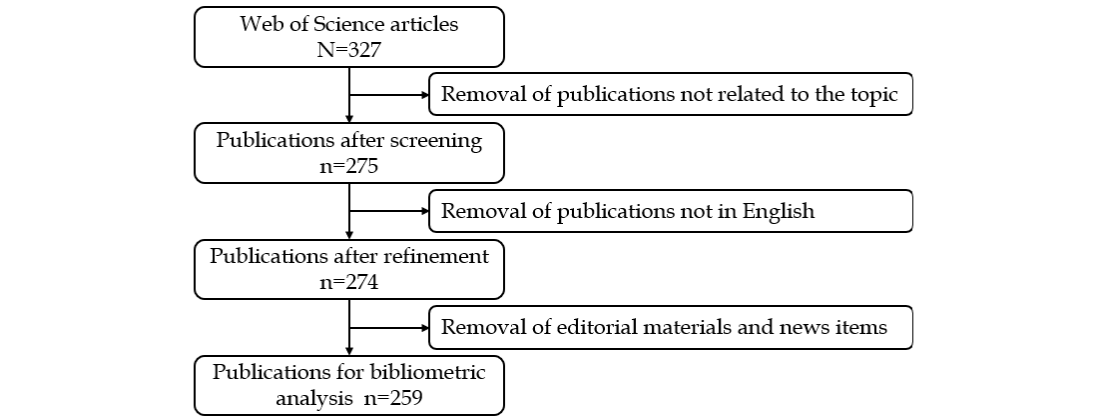
A diagram for the detailed selection criteria and bibliometric analysis steps of applying artificial intelligence to sepsis in the Web of Science Core Collection database.

The initial search yielded 327 articles focused on the application of AI in sepsis. After applying predefined inclusion criteria, 68 articles were deleted, leaving 259 articles for the final analysis (Figure 1).

**Figure 2:**
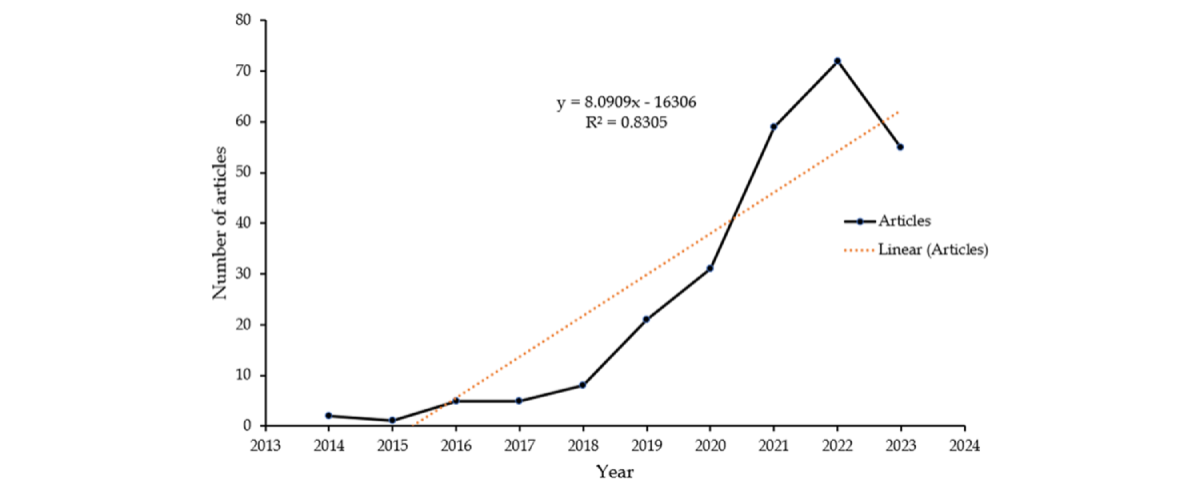
Trends in the number of publications on the application of artificial intelligence to the study of sepsis from 2014 to 2023 (August).

Over time, there has been a substantial rise in the number of publications in this field. Notably, the yearly publication number increased from just 2 articles in 2014 to 72 articles in 2022. It is important to note that before 2018, the yearly publication count did not cross 10 articles. The calculated annual growth rate was found to be 44.81% (Figure 2).

### Distribution of Source Journals

A total of 122 journals published articles on the application of AI in sepsis. Among them, the *Critical Care Medicine* journal was the most productive, having published 6.6% (17/122) of articles in this domain (Table 1). *Frontiers in Medicine*, *Scientific Reports*, and the *American Journal of Respiratory and Critical Care Medicine* were in the second, third, and fourth positions, publishing 17, 11, and 7 articles, respectively, on this topic. However, the top 10 journals published 86 articles, accounting for 33.2% (86/259) of all publications in this area.

**Table 1:** The top 10 journals with publications on the application of artificial intelligence in sepsis from 2014 to August 2023.

Rank	Journal	Country	Category	Publication frequency, n (%)	Impact factor in 2022	5-year impact factor
1	*Critical Care Medicine*	United States	Engineering, electrical, and electronics	17 (6.6)	8.8	8.4
2	*Frontiers in Medicine*	Switzerland	Multidisciplinary science	17 (6.5)	3.9	4.2
3	*Scientific Reports*	United Kingdom	Multidisciplinary science	11 (4.2)	4.6	4.9
4	*American Journal of Respiratory and Critical Care Medicine*	United States	Multidisciplinary science	7 (2.7)	24.7	21.9
5	*Frontiers in Immunology*	Switzerland	Clinical neurology	6 (2.3)	7.3	8.0
6	*Intensive Care Medicine*	United States	Neurosciences	6 (2.3)	38.9	27
7	*Journal of the American Medical Informatics Association*	United States	Computer science and interdisciplinary applications	6 (2.3)	6.4	6.3
8	*PLoS One*	United States	Neurosciences	6 (2.3)	3.7	3.8
9	*BMC Medical Informatics and Decision Making*	United Kingdom	Engineering and multidisciplinary	5 (1.9)	3.5	3.9
10	*Computers in Biology and Medicine*	United States	Engineering and biomedical	5 (1.9)	7.7	6.9

### Distribution of Countries and Regions

This study revealed that researchers from 73 countries and regions engaged in research on these subjects and published their work in various international peer-reviewed journals. Out of the total 259 articles, the United States made the most substantial contribution with 103 publications (39.8%), followed by China with 83 publications (32%), United Kingdom with 14 publications (5.4%), and Taiwan with 12 publications (4.6%) (Table 2).

**Table 2:** The top 10 countries and regions with publications on the application of artificial intelligence in sepsis from 2014 to August 2023.

Rank	Country	Articles, n (%)
1	United States	103 (39.8)
2	China	83 (32)
3	United Kingdom	14 (5.4)
4	Taiwan	12 (4.6)
5	India	11 (4.2)
6	Netherlands	10 (3.9)
7	Australia	8 (3.1)
8	Canada	8 (3.1)
9	Spain	7 (2.7)
10	Germany	7 (2.7)

### Distribution of Institutions

Table 3 shows the top 10 most productive institutes that used AI applications in sepsis. The University of California system (22/259 articles, 8.5%) ranked first among all research institutions, followed by Emory University (10/259 articles, 3.9%), Harvard University (10/259 articles, 3.9%), and Central South University (8/259 articles, 3.1%).

**Figure 3:**
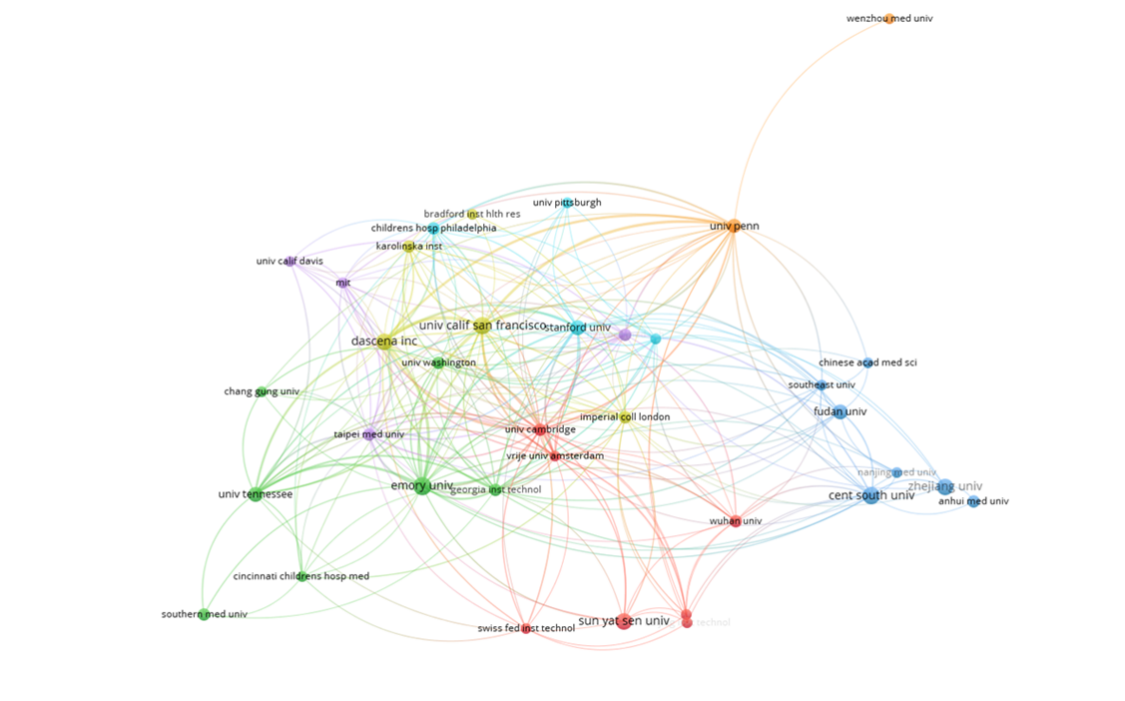
Institution co-operation network.

Figure 3 shows the institution cooperation network of 117 institutions that published at least 1 article.

**Table 3:** The top 10 institutes with publications on the application of artificial intelligence in sepsis from 2014 to August 2023.

Rank	Institutions	Country	Publications, n (%)
1	University of California system	United States	22 (8.5)
2	Emory University	United States	10 (3.9)
3	Harvard University	United States	10 (3.9)
4	Central South University	China	8 (3.1)
5	Dascena Inc	United States	8 (3.1)
6	University of Pennsylvania	United States	8 (3.1)
7	Zhejiang University	China	8 (3.1)
8	Stanford University	United States	7 (2.7)
9	Sun Yat-sen University	China	7 (2.7)
10	Fudan University	China	6 (2.3)

### Distribution of Authors

A total of 259 articles were authored by 1444 individuals, each of whom had at least 1 article to their name. In Table 4, we present the top 10 most prolific authors who conducted and published research in these fields. Ritankar Das secured the top position with 8 articles, closely followed by Chris Barton (6 articles), Rishikesan Kamaleswaran (6 articles), and Suchi Saria (6 articles).

**Figure 4:**
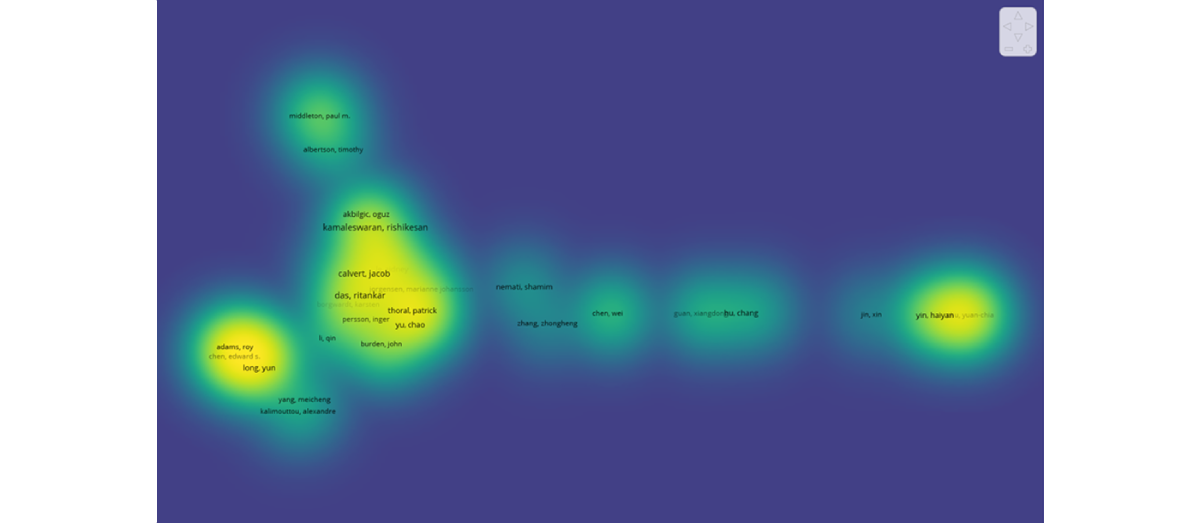
The co-authorship network of authors who contributed research on the application of artificial intelligence to sepsis from 2014 to 2023 (August).

Our analysis shows that 1444 authors have published at least 1 article. The largest set of associated authors consisted of 20 authors in 3 clusters (Figure 4).

**Table 4:** The top 10 authors with publications on the application of artificial intelligence in sepsis from 2014 to August 2023.

Rank	Author	Articles, n	Citations, n	h-index	Affiliation
1	Das	8	1417	18	Dascena Inc
2	Barton	6	2277	25	University of California San Francisco
3	Kamaleswaran	6	861	14	Emory University
4	Saria	6	2635	23	Johns Hopkins University
5	Calvert	5	1152	17	University of California Berkeley
6	Hoffman	5	957	14	Dascena Inc
7	Li	5	2	1	Sun Yat-sen University
8	Nemati	5	2402	23	University of California San Diego
9	Adams	4	118	6	Johns Hopkins University
10	Davis	4	1253	17	University of Tennessee Health Science Center

### Articles Cocitation Analysis

Table 5 shows the top 10 most frequently cited publications. The publication that received the most citations was by Komorowski et al [16], titled “The Artificial Intelligence Clinician learns optimal treatment strategies for sepsis in intensive care,” published in *Nature Medicine* in 2018 and received a total of 408 citations as of August 31, 2023.

**Table 5:** Top 10 cited articles in the application of artificial intelligence on sepsis research from 2014 to August 2023.

Rank	Author	Journal	Title	Citation, n
1	Komorowski et al [16]	*Nature Medicine*	The Artificial Intelligence Clinician learns optimal treatment strategies for sepsis in intensive care	408
2	Nemati et al 2018 [17]	*Critical Care Medicine*	An Interpretable Machine Learning Model for Accurate Prediction of Sepsis in the ICU	329
3	Taylor et al 2016 [18]	*Academic Emergency Medicine*	Prediction of In-hospital Mortality in Emergency Department Patients With Sepsis: A Local Big Data-Driven, Machine Learning Approach	257
4	Desautels et al 2016 [19]	*JMIR Medical Informatics*	Prediction of Sepsis in the Intensive Care Unit With Minimal Electronic Health Record Data: A Machine Learning Approach	226
5	Fleuren et al [10]	*Intensive Care Medicine*	Machine learning for the prediction of sepsis: a systematic review and meta-analysis of diagnostic test accuracy	184
6	Horng et al 2017 [20]	*PlosOne*	Creating an automated trigger for sepsis clinical decision support at emergency department triage using machine learning	148
7	Gultepe et al 2014 [21]	*Journal of the American Medical Informatics Association*	From vital signs to clinical outcomes for patients with sepsis: a machine learning basis for a clinical decision support system	101
8	Giannini et al 2019 [22]	*Critical Care Medicine*	A Machine Learning Algorithm to Predict Severe Sepsis and Septic Shock: Development, Implementation, and Impact on Clinical Practice	100
9	Hou et al 2020 [23]	*Journal of Translational Medicine*	Predicting 30-days mortality for MIMIC-III patients with sepsis-3: a machine learning approach using XGboost	98
10	Mani et al 2014 [24]	*Journal of the American Medical Informatics Association*	Medical decision support using machine learning for early detection of late-onset neonatal sepsis	97

### Co-Occurrence Analysis of Top 100 Keywords

Keywords encapsulate the central themes within a publication and are ideal for examining interconnected areas of research. In this study, we performed co-occurrence analysis to pinpoint the prominent research focal points in the field of AI application in sepsis research, using the top 100 keywords. The extraction and clustering of these top 100 keywords were performed using VOSviewer.

**Figure 5:**
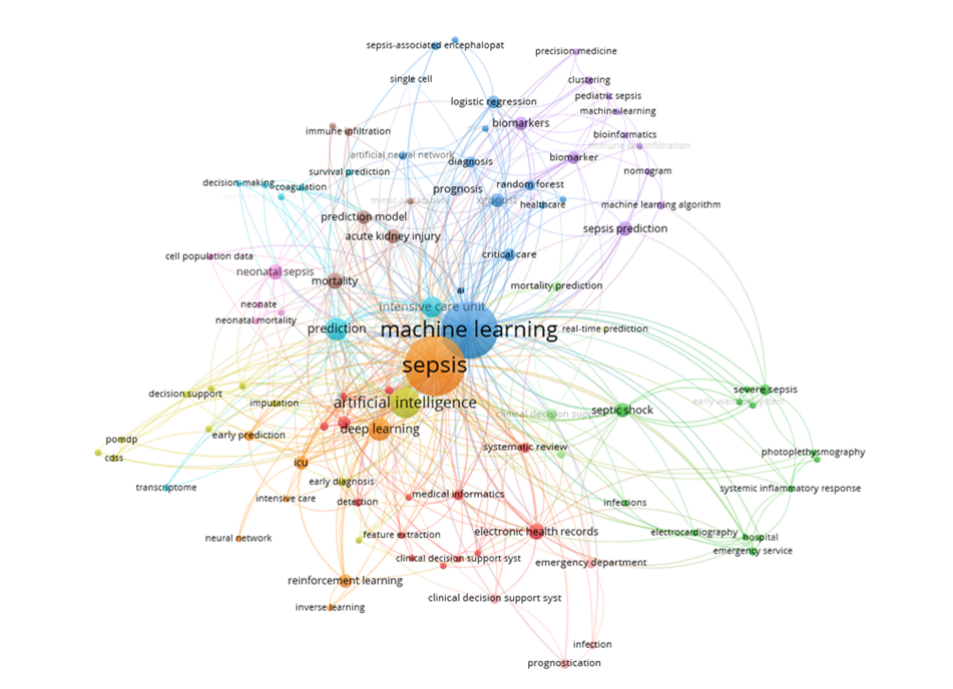
The co-occurrence network of the top 100 keywords in artificial intelligence research for sepsis from 2014 to 2023 (August).

Figure 5 illustrates our use of VOSviewer to create a visual network map, consisting of 6 clusters based on the co-occurrence of the top 100 keywords. The core of this visualization network map is occupied by the following keywords: sepsis (n=138), machine learning (n=122), artificial intelligence (n=35), and deep learning (n=20).

## Discussion

### Main Findings

This study investigated the citation analyses of AI models in sepsis research, scrutinizing the publication patterns related to their application. This study reports a noteworthy upswing in interest in this subject over the past decade, particularly increasing from 2018 to 2022.

The substantial increase in the use of AI in health care research is noteworthy. For instance, there was an 88.88% increase in the use of AI in health care research after 2012, with only 10 countries contributing to over 96% of the studies [25]. Our findings also indicate an annual growth rate of 44.81% in the use of AI in sepsis-related research. If this growth rate continues in the future, we can anticipate the publication volume in the domain of AI in sepsis-related research doubling approximately every 5 years. This increased number of publications indicates advancements, improved functionality, and the overall progress of AI in sepsis, especially when compared to other application areas.

The number of publications exhibits geographical disparities [26-28]. Recent research indicates a rise in the application of AI in health care research, particularly in developed countries [12,29,30]. These nations are investing increased funds into AI research and the development of AI tools to improve health outcomes. This study aligns with these findings, highlighting an increased number of publications originating from developed countries. However, it underscores the importance of researchers from developing nations stepping forward to contribute toward achieving ultimate health goals. Effective collaboration among clinical experts, AI model developers, and health care providers is essential to addressing the challenges at hand [31].

Selecting the appropriate journals for publication can be a complex decision [32]. Authors take various factors into account when submitting, such as the accessibility of open-access journals and the higher impact factor associated with certain subscription journals [33]. Some authors prefer open-access journals for their widespread availability after publication and the potential for a higher number of citations [34]. Conversely, subscription journals from reputable publishers attract attention due to their high impact factors [35,36]. New researchers often struggle with the dilemma of where to submit their work. This study shows that authors frequently weigh both open-access and higher-impact–factor journals when publishing research related to the application of AI in sepsis.

This study aimed primarily to highlight the most commonly used data sets and algorithms in the current literature. The majority of studies used the Multiparameter Intelligent Monitoring in Intensive Care III (MIMIC-III) data set and supervised machine learning models [37-39]. Notably, Komorowski et al [16] introduced a reinforcement learning model to predict sepsis in patients, demonstrating average reliability levels higher than those of human clinicians. While many studies used extensive training and testing data sets, the majority focused on single-centered data [39]. To apply these models in real-world clinical settings, external validation becomes necessary.

### Strengths and Limitations

This study has several strengths. First, it is the first comprehensive bibliometric analysis that sheds light on the research trends of the application of AI in sepsis, illustrating how this field has evolved. Second, this study gauges productivity in terms of sources, authors, institutions, and countries, while also visualizing word trends. This provides novel and in-depth insights for both researchers and practitioners. This study also has some limitations to address. First, we only collected relevant publications from the Web of Science, a widely used academic resource, for bibliometric analyses [13,40-42]. Nevertheless, using other databases, such as PubMed or Scopus, might have provided slightly varied findings. Second, our inclusion criteria comprised articles published solely in English. However, inclusion of other languages, gray literature, and books might have influenced outcomes, particularly considering diverse cultural perspectives among scholars on the application of AI in sepsis. Finally, relying solely on article titles for the search may pose limitations. However, our aim was to focus on publications specifically addressing the application of AI in sepsis. Therefore, a title screening was deemed more suitable than a broader topic search.

### Conclusion

This study aimed to present a comprehensive overview of the use of AI in sepsis through a systematic analysis of existing literature. The findings of this study reveal a noticeable increase in the number of publications over the last 10 years. Until now, developed countries have been the primary contributors in this field. Researchers from developing countries should step forward, leveraging population advantages and core technologies in different regions to foster collaboration.

Leading multidisciplinary science journals, including *Frontiers in Medicine, Scientific Reports*, and the *American Journal of Respiratory and Critical Care Medicine*, emerge as key contributors to this topic based on the volume of published articles. As the application of AI in sepsis research continues to rise, this study serves as a valuable resource for researchers seeking direction and opportunities for collaboration.

## Abbreviations

AI: artificial intelligence
MIMIC-III: Multiparameter Intelligent Monitoring in Intensive Care III
